# Identification of Potential Lead Compounds Targeting Novel Druggable Cavity of SARS-CoV-2 Spike Trimer by Molecular Dynamics Simulations

**DOI:** 10.3390/ijms24076281

**Published:** 2023-03-27

**Authors:** Yizhen Zhao, Yifan Zhao, Linke Xie, Qian Li, Yuze Zhang, Yongjian Zang, Xuhua Li, Lei Zhang, Zhiwei Yang

**Affiliations:** 1 MOE Key Laboratory for Nonequilibrium Synthesis and Modulation of Condensed Matter, School of Physics, Xi’an Jiaotong University, Xi’an 710049, China; 2 School of Life Science and Technology, Xi’an Jiaotong University, Xi’an 710049, China

**Keywords:** spike trimer, in situ full-length structure, dynamic conformational changes, molecular dynamics simulations, SARS-CoV-2 inhibitors

## Abstract

The global pandemic of severe acute respiratory syndrome coronavirus 2 (SARS-CoV-2) has become an urgent public health problem. Spike (S) protein mediates the fusion between the virus and the host cell membranes, consequently emerging as an important target of drug design. The lack of comparisons of in situ full-length S homotrimer structures in different states hinders understanding the structures and revealing the function, thereby limiting the discovery and development of therapeutic agents. Here, the steady-state structures of the in situ full-length S trimer in closed and open states (S^closed^ and S^open^) were modeled with the constraints of density maps, associated with the analysis of the dynamic structural differences. Subsequently, we identified various regions with structure and property differences as potential binding pockets for ligands that promote the formation of inactive trimeric protein complexes. By using virtual screening strategy and a newly defined druggable cavity, five ligands were screened with potential bioactivities. Then molecular dynamic (MD) simulations were performed on apo protein structures and ligand bound complexes to reveal the conformational changes upon ligand binding. Our simulation results revealed that sulforaphane (SFN), which has the best binding affinity, could inhibit the conformational changes of S homotrimer that would occur during the viral membrane fusion. Our results could aid in the understanding of the regulation mechanism of S trimer aggregation and the structure-activity relationship, facilitating the development of potential antiviral agents.

## 1. Introduction

Severe acute respiratory syndrome coronavirus 2 (SARS-CoV-2), has continuously spread to 219 countries and territories, with increased global health concerns (https://covid19.who.int, accessed on 8 November 2022). The receptor binding domain (RBD) of spike protein (S) is responsible for binding to the host cell receptors (e.g., angiotensin-converting enzyme 2, ACE2) to facilitate membrane fusion and virus entry, making it a promising target for the development of vaccine/antibody candidates [1]. SARS-CoV-2-neutralizing antibodies have shown protective efficacy, but the rapid emergence of new SARS-CoV-2 variants could reduce the effectiveness [2,3,4,5,6]. Low molecular weight compound may be an effective way to prevent the viral attachment and infection. In fact, some compounds that interact with the S RDB, receptor ACE2, and RBD-ACE2 binding interface have shown antiviral potential, such as arbidol, CPD7, and polymerase inhibitor SPC-14 [7,8,9]. However, a number of various mutations have been reported in the binding pocket of RBD (located between S338 to Q506), which are responsible for the altered interactions with human receptors, resulting in resistance to existing vaccines, antibodies, and agents [10].

**Figure 1:**
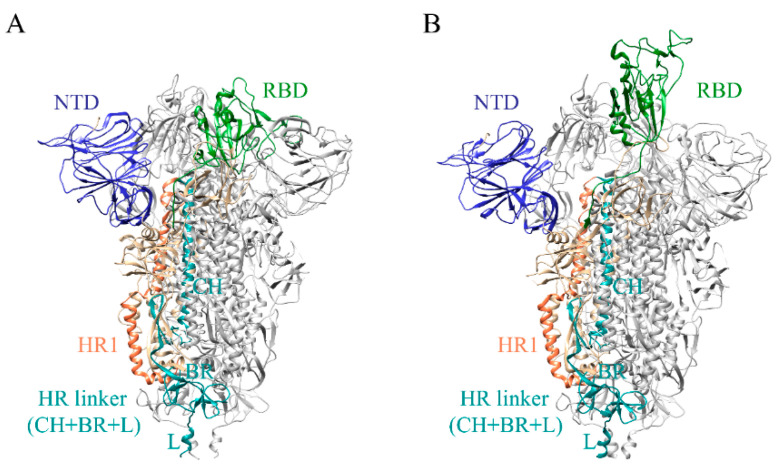
Schematic representation of SARS-CoV-2 S protein in (**A**) closed state (S^closed^, PDB ID: 7DDD) and (**B**) open state (S^open^, PDB ID: 7DDN). N-terminal domain (NTD), receptor-binding domain (RBD), heptad repeat1 (HR1), and HR linker were colored by blue, green, coral, and dark cyan, respectively.

Spike protein is a trimeric glycoprotein located on the SARS-CoV-2 surface, and each monomer consists of two subunits, S1 and S2. The former includes N terminal domain (NTD), receptor binding domain (RBD), C-terminal domain (CTD); the latter includes fusion peptide (FP), heptad repeat 1 (HR1), HR linker (CH: central helix + BR: β-rich region + L: linker), heptad repeat 2 (HR2), transmembrane (TM), and cytoplasmic tail (CT) (Figure 1A,B). Two proteolytic cleavage sites, including S1/S2 and S2′, trigger a larger structural transition of S trimer [11]. In the prefusion state, the RBD of S trimer in closed state (S^closed^) rotates upward with counterclockwise rotation of the CH region resulting in open conformation (S^open^) for binding to the cellular receptor (Figure 1) [12]. Subsequently, the S trimer changes to the postfusion state with the loss of the NTD and the formation of the six-helical bundle (6-HB) facilitated by the interactions between HR1 and HR2 [13,14]. The conformational changes of S trimer induce the viral entry and infection, thus the inhibition of the process might be a new approach for drug design [15].

Current studies suggest that inhibiting the formation of six-helix bundle core (6-HB) should be effective against SARS-CoV-2 entry [16]. Arbidol (available anti-influenza drug) has been used for the treatment of SARS-CoV-2 infection by targeting the S trimers and impeding the trimerization [7]. Two clinically approved drugs, ITZ and EB, interact with the HR1 region to inhibit viral entry and present a mechanism of resistance to neo-coronavirus infection in vitro [13]. Ursodeoxycholic Acid (UDCA) has a strong affinity with the CH region (residues K986 to C1036) of S trimers, preventing conformational changes upon binding to ACE2 [17]. Considering only a limited number of antivirals have been approved for the treatment of SARS-CoV-2 infection, it is necessary to continue the search for potential agents based on the conformational changes of S trimers.

Due to the limitations of the cryo-electron tomography (cryo-ET) method and the high flexibility of the stalk region which connects S trimer with the viral membrane, current resolved S trimer structures have low resolution and miss some regions, making comparisons of the full-length structures in different states and the analysis of the conformational transition confusing. In this study, we established and optimized the in situ full-length S structures both in closed and open states (S^closed^ and S^open^). Through the comparative structure analysis, the key sites for conformational changes were determined and used for the subsequent docking and molecular dynamics (MD) simulations with bioactive compounds of ZINC database [18]. Five lead compounds were selected, and the binding profiles of the most potential compound with S^closed^ and S^open^ structures were evaluated by structural analysis and the molecular mechanics generalized born surface area (MM/GBSA) method. In addition, the dynamic changes of S trimers upon the ligand binding were analyzed by the principal component analysis (PCA) and dynamic cross-correlation matrices (DCCM) estimation. Our results could aid in the understanding of conformational alteration of S trimers and could be beneficial in developing antiviral drugs targeting the changes of dynamic structures.

## 2. Results and Disussion

### 2.1. Full-Length S Structure Model

**Figure 2:**
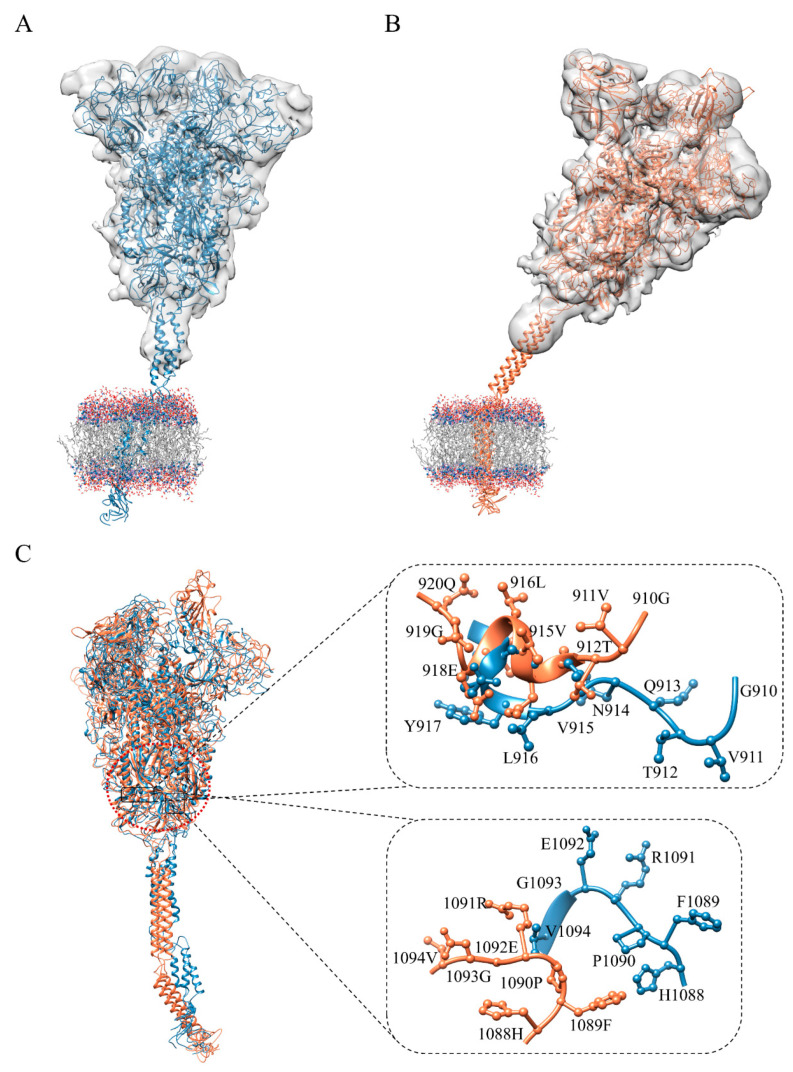
The structures of in situ S trimer in the closed (S^closed^, **A**) and open states (S^open^, **B**), and the structure differences of the sequence 910–920 in the HR1 domain and sequence 1088–1094 in the HR linker between S^closed^ and S^open^ structures (**C**). TM domain of S trimer was embedded into the bilayer membrane (molar ratio of POPC:cholesterol = 9:1) with the EM density maps fitting. The S^closed^ and S^open^ structures were respectively colored by blue and coral.

Given the impact of structural integrity on ligand bindings, full-length SARS-CoV-2 S^closed^ and S^open^ structures were constructed based on the reported cryo-electron microscopy structures (7DDD and 7DDN) [19]. Firstly, missing loops in the N-terminal region (residues 1–13), RBD (residues 70–76, 248–254), and CTD (residues 621–640 and 671–688) were added by Discovery Studio [20]. In accordance with previous reports of S trimer modeling [21,22,23], HR2 domain (residues 1159–1211), TM domain (residues 1212–1233), and CT domain (residues 1234–1273) were subsequently constructed by using the templates of SARS-Coronavirus HR2 domain (PDB ID: 2FXP) [24,25], transport protein (PDB:7KAL) [26], and metallothionein-2 (PDB: 1MRT) [27], associated with the 96%, 32% and 50% sequence identities, respectively. In accord with the cryo-ET and molecular dynamics (MD) simulation results, the stalk of S trimer exhibits visible hinging motions [22], hence the transmembrane domains of S^closed^ and S^open^ structures were embedded into the bilayer membrane (molar ratio of POPC:cholesterol = 9:1) with the angle of inclination 40° [28] (Figure 2A,B). The obtained S^closed^ and S^open^ structure models were further optimized utilizing the 100-ns MD simulations (Figure S1).

Previous cryo-ET studies have determined the S structure with conformational flexibility on the virion surface. To obtain the more reasonable full-length S structures in terms of C3 symmetry constraint and orientation of each part of the stalk, we fitted the S^closed^ and S^open^ structure models into cryo-ET density maps (EMDB access code: 11494 and 11495) [29]. Molecular dynamics flexible fitting (MDFF) was adopted to ensure stereochemical accuracy and preservation of secondary structure and other structural features [30]. Potential energy proportional to the electron density map is introduced to provide local constraints on the structure [21]. The final structures of S^closed^ and S^open^ were checked by Procheck program [31] and Profile-3D program [20], associated with 90% residues being allowed in region of Ramachandran plot (Figure S2A,B) and 90% residues exhibiting reasonable folding (Figure S2C,D).

The N-terminal of HR1 region (910–920) is mostly aliphatic amino acids; the secondary structures of that are α helices and loops. The residues 1088–1094 located in the HR linker region consist of aliphatic, heterocyclic and aromatic amino acids with more non-polar amino acids including Phe1089, Pro1090, Gly1093, and Val1094 in this region, indicating that the sequence is closed to the hydrophilic region outside the trimer with a crowd of uncharged amino acids (Figure 2C). It is well-known that α helix structure has strong rigidity via hydrogen bonds, which is beneficial to maintain protein conformation, while loop structure can change its direction flexibly. RBD of S^open^ is continuously supported to ensure an upward state (Figure 1B) for binding to the receptor, which explains why it contains many α helices in the polymerization site of the trimer. More irregular curls in the closed conformation (S^closed^) than that in open conformation (S^open^) may be related to the structure transition from closed state to open state. The flexibility of the HR1 and HR linker facilitates the transition of RBD to the upward state and enables the S trimer to be receptor accessible. Therefore, ligands binding to this region promisingly block structural alterations. Meanwhile, due to the low variability, the trimer cavity formed by HR1, CH, and CD (from A, B and C chains of S trimer) has been used as potential drug target for virtual screening [32,33]. Chitosan, macrolide types and phthalocyanine derivatives can inhibit the conformational changes induced by SARS-CoV-2 entry into target cells through the interactions between the surrounding regions of the trimer cavity [32,33]. Considering the effect of the flexible stalk of S protein on structural changes and the vital role in the membrane fusion process, the defined trimeric cavity region was expanded to use as a binding pocket for drug screening (Figure 2C).

### 2.2. Docking Hits

We employed virtual screening approaches targeting the pockets formed by amino acids near HR1 and HR linker of full-length S structures in closed and open states to discover potential anti-SARS-CoV-2 molecules from bioactive compounds of ZINC database [34] by using the LibDock method [35]. The top-five ligands, which have high assessment scores (above 58) and underlying therapeutic effects, were selected for further analysis (Table 1). Among them, sulforaphane (SFN: 1-isothiocyanate-4-methyl sulfonyl butane) (ZINC000002557133) belonging to the class of isothiocyanates is a natural phytochemical existing mainly in cruciferous vegetables such as broccoli [36]. SFN has antioxidative and anti-inflammatory effects and is an activator of nuclear factor erythroid 2 related factor 2 (Nrf2), which is a key transcription factor that regulates resistance to oxidative stress. In addition, SFN shows anticarcinogenic characteristics through providing protection against certain carcinogens and toxic, reactive oxygen species by inducing phase II detoxification enzymes [36,37]. Interestingly, in vitro cellular experimental studies have shown that SFN can inhibit the gene expression of IL-6 and IL-8 induced by the SARS-CoV-2 S protein evaluated by RT-qPCR and Bio-Plex analysis [38]. Isometheptene (ZINC000001683250) is an effective sympathomimetic drug for acute migraine treatment [39]. Urocanic acid (ZINC000034633903), an imidazole-acrylic acid derivative, has immunosuppressive properties in skin and systemic diseases and plays a part in acidifying the cytosol of tumor cells [40]. 1-Octanol (ZINC000001532735) has tremor suppression properties, anti-inflammatory, and analgesic effects [41]. Allicin (ZINC000004097409) isolated from *Allium chinense*, *Allium nutans*, and other organisms is beneficial to the cardiovascular system and has antifungal action [42]; it also shows certain inhibitory effects on the SARS-CoV-2 virus replication [43].

**Figure 3:**
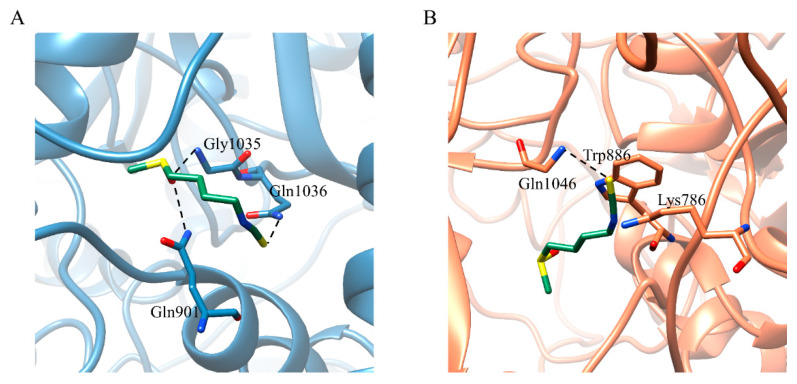
Detailed view of SFN binding to the (**A**) S^closed^ and (**B**) S^open^. The key residues are represented by stick models, and the important H-bonding interactions are labeled in the black lines. The C atoms of S^closed^ and S^open^ are colored in blue and coral, respectively. The O, N, S atoms are colored in red, blue and yellow, respectively.

SFN has H-bonding interactions with residues Gly901, Gly1035, and Gln1036 of C chain (Gly901/C, Gly1035/C and Gln1036/C) in the SFN-S^closed^ complex (Figure 3A). Isometheptene and Allicin bind S by the hydrophobic interactions with Arg685/A, and Val781/C, Ala1025/C, and Ala1026/C (Figure S3A,D). The carboxyl of urocanic acid forms the hydrogen bond with Thr874/C (Figure S3B). The hydroxyl of 1-octanol has the H-bonding interaction with Arg685/A, and 1-octanol forms hydrophobic interactions with Tyr873/C, Pro1053/C, and Val1060/C (Figure S3C). Regarding the S^open^ in complexes with five potential agents, SFN has the H-bonding interaction with residue Gln1046/B, electrostatic interactions with residues Lys786/C and Trp886/C (Figure 3B). Isometheptene only has the hydrophobic interactions with hydrophobic residues Val729/A, 781/A, 785/A, 1060/A, Ty873/A, and Ala1025/A, 1026/A, wherein the Val781/A, Ala1025/A, and Ala1026/A are involved in the hydrophobic effects of isometheptene-S^closed^ complex (Figure S4A). Residues Ala684–Val687/A and Val785/B, Leu877/B, Leu1034/B are responsible for the binding between S^closed^ and two ligands urocanic acid, 1-octanol. The atom O of both ligands possesses the H-bonding interactions with residue Ser686/A and the two ligands also form the hydrophobic interactions with Leu877/B and Leu1034/B (Figure S4B,C). Allicin has the H-bonding interactions with residues Ser704/B and Thr887/C and exits the hydrophobic interactions with residues Pro1069 and Val1094 (Figure S4D).

### 2.3. Binding Free Energy between SFN and S Trimer

**Figure 4:**
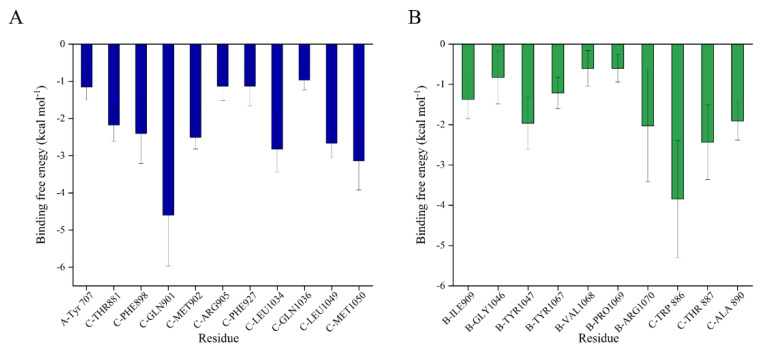
Average contribution of the residues to the binding free energies (Δ*G_bind_*) within the (**A**) SFN-S^closed^ and (**B**) SFN-S^open^ complexes. Per-residue free energy decomposition was conducted by the MM/GBSA method implemented in AmberTools18 [46,47].

Considering the results of virtual screening and the potential efficacious functional groups, the SFN-S^closed^ and SFN-S^open^ complexes were selected for the 200ns MD simulations. As shown in Figure S5A,B, the time evolutions of backbone-atom RMSDs indicated that the S^closed^, S^open^, SFN-S^closed^, and SFN-S^open^ complex systems reached the fundamental convergence during the last 120 ns (80–200 ns), which were used for the subsequent energy analysis. SFN significantly reduces the S^closed^ flexibility overall and stabilizes the certain regions of the S^open^ (Figure S5C,D). The binding free energies (Δ*G_bind_*) of SFN with S^closed^ and S^open^ are summed to −22.27 ± 1.12 and −14.21 ± 2.53 kcal mol^−1^, respectively (Table 2). Besides, the binding contribution of each individual residue in the docked complexes were evaluated, and residues with contributions of less than −0.5 kcal mol^−1^ were collected in Figure 4. SFN is mainly in contact with the sTM linking the FP and HR1 regions (residues Thr881, Phe898, Gln901, Met902, Arg905/C), HR1 region (residue Phe927/C) and HR linker region (residues Leu1034, Gln1036, Leu1049, Met1050/C) of S^closed^, and most of these interactions are consistent with above structural analysis (Figure 3A). Residues Gly1046, Tyr1047, Tyr1067, Val1068, Pro1069, and Arg1070 in the HR linker of B chain and residues Trp886, Thr887, and Ala890 in the sTM of C chain were found to contribute to the binding between SFN and S^open^ (Figure 4B).

SFN interacts with a wide range of S^closed^ including sTM, HR1, CH, and other regions of the HR linker, which undergo structural rearrangement with the extension and flip approximately 180° of HR1 during the fusion process. SFN mainly contacts with some residues of sTM and HR linker of S^open^. In the current studies, small molecules screened for the trimer cavity mainly interact with the HR1 region because the structure near HR1 and CH regions exhibits a high degree of evolutionary conservation in β-coronavirus compared to RBD and NTD of S1 subunit [44]. For example, the molecules phthalocyanine, hypericin, troxerutin, thymopentin, and daclatasvir interfere with the entry of the SARS-CoV-2 by forming hydrogen bonding interactions or hydrophobic interactions with certain residues of HR1 and HR linker region, blocking the movement of the regions near HR1 to interfere with the entry of the SARS-CoV-2 [33,45]. All these results support the hypothesis that the presence of these drugs in the pocket may block the region near HR1 and CH and prevent the large S conformational changes that allow SARS-CoV-2 virions to enter the target cells.

### 2.4. Conformational Changes upon Ligand Binding

**Figure 5:**
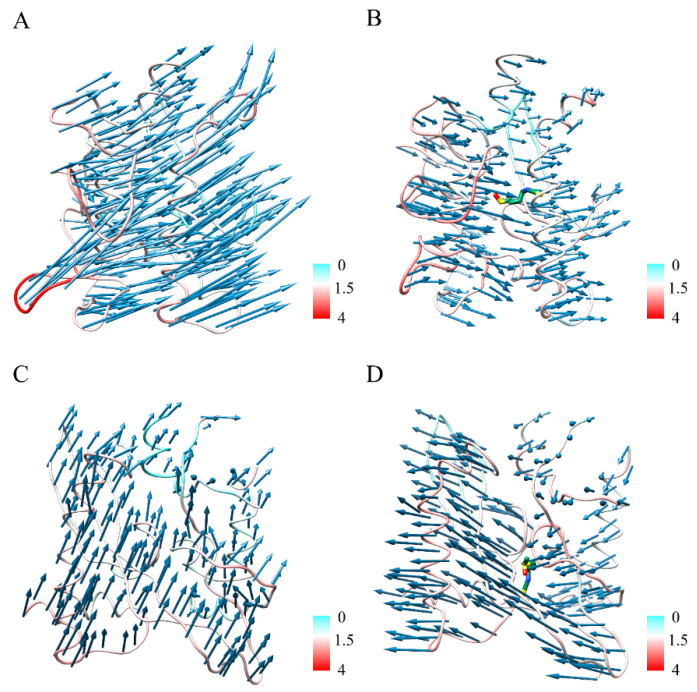
Vector field representations of the first principal component (PC) obtained in the (**A**) S^closed^, (**B**) SFN-S^closed^, (**C**) S^open^, and (**D**) SFN-S^open^ systems. The colors of the residues indicate the root-mean-square fluctuation (RMSF) values (units in Å).

We noted dynamic changes in the protein structure near the small molecule (Ser680–Val722/A, Gln784–Val826/C, Ile870–Ser943/C, and Ser1030–Val1068/C) by PCA analysis, and Figure 5 shows the fluctuation of the binding pocket along the trajectory and the trend of PC1 movement in the S^closed^ and SFN-S^closed^ systems, respectively. Without the ligands, S showed a clear counterclockwise rotational trend, which is favorable to the instantaneous raising up of the RBD [12] (Figure 5A), while the binding of SFN inhibited the flexibility of the binding pocket and some amino acids in the vicinity including the cleavage site S1/S2, FP, sTM, HR1, and CH regions, attenuating their motility trends (Figure 5B). The amino acids near the binding pocket (Gln774–Lys790/C, Ile870–Gly910/C, Gln690–Pro728/A, Pro897–Ala944/A, and Ser1037–Ala1080/A) of apo S^open^ system were less flexible relative to the apo S^closed^ structure and had less pronounced motility tendency (Figure 5C), but the binding of SFN changed its original direction of motion and may inhibit its binding to the receptor (Figure 5D). The ligand SFN stabilizes the protein structure near FP and HR1 by forming H-bonding, hydrophobic, electrostatic interactions and alters the movement trend of the structure of S^open^ binding pocket, and potentially inhibits both the structural transformation of S proteins from closed state to open state and the structural changes from open state to the postfusion state, thereby preventing membrane fusion of the virus with the host cell.

**Figure 6:**
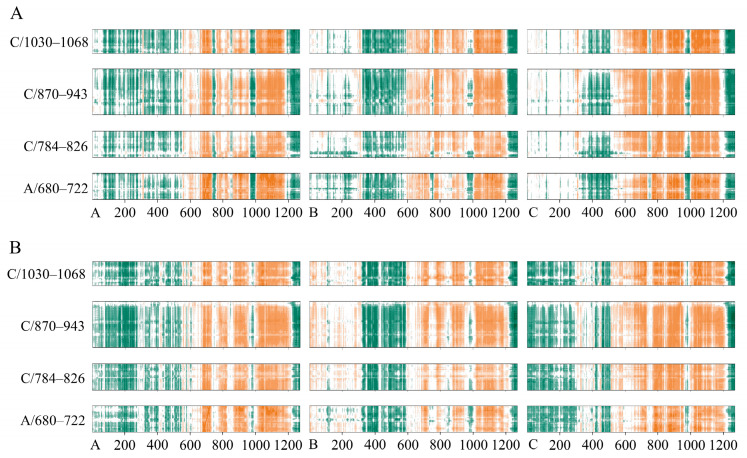
Dynamic cross-correlation maps for S in the (**A**) S^closed^ simulation and (**B**) SFN-S^closed^ simulation. Correlation values range from −1 to +1; the positive values (sea green) indicate that 2 residues are correlated and negative values (orange) indicate that they are anti-correlated.

**Figure 7:**
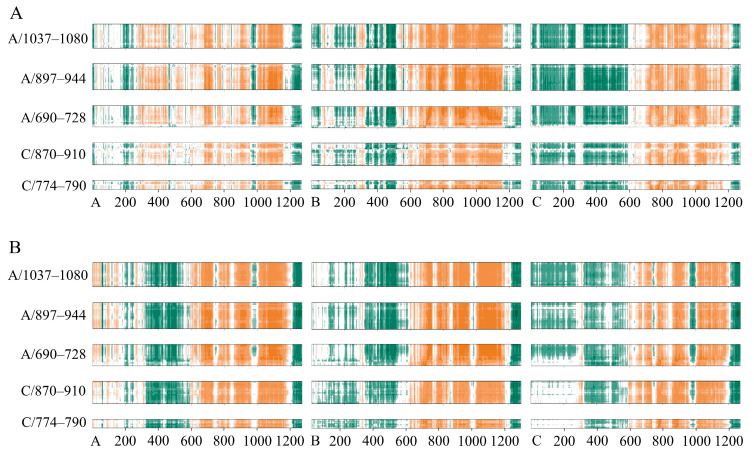
Dynamic cross-correlation maps for S in the (**A**) S^open^ simulation and (**B**) SFN-S^open^ simulation. Correlation values range from −1 to +1; the positive values (sea green) indicate that 2 residues are correlated and negative values (orange) indicate that they are anti-correlated.

The structural dynamics of S^closed^ and S^open^ induced by SFN were further analyzed by the dynamic cross-correlation map or matrix (DCCM). In the DCCM plot, orange indicates significant positive correlation, dark green indicates obvious anti-correlation, while white regions indicate less correlation. For the convenience of analysis, only the correlations of residues Ser680–Val722/A, Gln784–Val826/C, Ile870–Ser943/C, and Ser1030–Val1068/C near the binding pocket of S^closed^ are shown. The binding of SFN to S^closed^ enhanced the anti-correlation of residues within the NTD regions of the A, especially C chains, and the anti-correlation with NTD region of B chain changed to positive correlation. SFN weakened the anti-correlation of residues near the binding pocket within RBD regions (331–527) of A and C chains. The extent of the anti-correlation between residues in the binding pocket and residues 970–998 located in the HR1 and CH regions of three chains was reduced by the binding of SFN. The degree of positive correlation of residues in the binding pocket with HR linker region and some amino acids in HR2 (1015–1188) of C chain was slightly weakened, probably due to the H-bonding interactions of SFN with S^closed^ stabilizing this region (Figure 6A,B). We analyzed the correlations of the residues Gln774–Lys790/C, Ile870–Gly910/C, Gln690–Pro728/A, Pro897–Ala944/A, and Ser1037–Ala1080/A with the S^open^. The green region showed the anti-correlation between regions near FP (774–790) and sTM (870–910) with NTD, which is attenuated or even disappeared of the C chain. The RBD regions of A and B chain had strong anti-correlation values with the binding pocket region following the regulation of SFN and the correlation of the A chain was flipped, while the negative correlation between these two amino acid segments of C chain with RBD up was instead diminished. Obviously, the binding pocket shows enhanced positive correlation with most of the structures of S2, including FP, HR1, and HR2 in the three chains, but a significant negative correlation with the partial amino acids (978–1005) of HR1 and CH of C chain (Figure 7A,B). The RBD upward is necessary for S trimer binding to the cellular receptors for membrane fusion. SFN alters the dynamic cross-correlation of S^open^, especially the binding pocket and the region responsible for the extension of the structural transition from prefusion to postfusion, which may inhibit the conformational changes of S^open^ and suppress the interaction of FP and TM with the membrane [48].

Based on the conformation motion between the RBD and the subdomain 1 (SD1)–subdomain 2 (SD2) junction, Qian Wang et al. designed the compound CPD7 using a structure-based virtual screening approach, which can insert between SD1 and SD2 of the S trimer to impede RBD opening to the up state, and its inhibition effect was experimentally evaluated by RT-PCR and SPR methods [8]. In addition, the cryo-EM structure revealed that compound SPC-14 could shift the conformation of S trimer toward the closed state [9]. Recently, the work of Carla Zannella et al. further proved that this approach is reliable. The docking results revealed that two peptides, TLH and VFI, can bind to pockets within the S1 and S2 domains; in vitro experiments demonstrated their inhibiting effects on SARS-CoV-2 infection [49]. Most importantly, Jessica Gasparello et al. showed that exposure of epithelial IB3-1 cells to SARS-CoV-2 S protein induced increased release, particularly of cytokines/chemokines causing the deep inflammatory state, while previous cell experiments have revealed that SFN might reverse S protein-induced upregulation of IL-6 and IL-8, showing certain therapeutic effects on viral infections [38]. In addition, experiments to determine the effects of SFN on toxicity and apoptosis showed that SFN-based treatment had little effect on cell growth and did not induce apoptosis. Based on at least the above results, SFN is likely to be a potential antiviral agent.

## 3. Materials and Methods 

### 3.1. In Situ Full-Length S Trimer Structure Modeling

The initial coordinates of S trimer in closed and open states (S^closed^ and S^open^) were retrieved from Protein Data Bank (accession code: 7DDD and 7DDN) [19]. The missing loops, HR2, transmembrane domain (TM), and unstructured C terminal region (CT) of S^closed^ and S^open^ structures were added using Discovery Studio [20], based on the full-length amino acid sequence (Uniprot ID: P0DTC2). The missing hydrogen atoms were added with the expected protonation states of residues [50]. Each model was inserted into a palmitoyl-oleoyl-phosphatidyl-choline POPC/cholesterol (9:1 molecular ratio) bilayers [51] using the CHARMM-GUI Membrane Builder [52], in accordance with the orientation of S trimers on the viral membranes revealed by cryo-ET results [22]. The two structures were geometry-optimized using the conjugate gradient (CG) method [53], and further refined by 100 ns molecular dynamics (MD) simulations using AMBER18 software [54,55]. Protein and POPC/cholesterol bilayers were described using AMBER ff14SB [56] and Lipid17 [57] force fields, which have been routinely applied for MD simulations of various membrane protein systems [58].

### 3.2. Molecular Dynamics Flexible Fitting (MDFF)

To obtain better and more accurate full-length S structures, the molecular dynamics flexible fitting procedure (MDFF) was performed to flexibly fit the protein structures into the EM density maps (EMDB access code: 11494 and 11495) [29]. S^closed^ and S^open^ systems were simulated for 5 ns with a scaling factor of 0.3 using the NAMD2 [30,59]. The protein atoms moved towards the high-density regions of the map and preserved the secondary structure elements during MDFF fitting [60,61].

### 3.3. Docking with Multiple Conformations

Virtual screening process was performed on the full-length structures of S^closed^ and S^open^ using the LibDock approach by aligning adequately generated ligand conformations to protein site features (HotSpots) [62]. The structures and partial atomic charges of used compounds (bioactive compounds of ZINC database [18]) were handled by the “Minimize Ligands” tools [20]. The details of receptor-based screening agree with our previous works [63]. Briefly, the binding sites of receptors were assigned with a sphere of 10.0 Å, and the optimal orientations of compounds within proteins were probed on the basis of interactions with binding residues and geometrical matching qualities. The optimal docked complexes were further selected to be energy-minimized using the conjugate gradient (CG) method, until converged to 0.01 kcal mol^−1^ Å^−1^.

### 3.4. Molecular Dynamics (MD) Simulations

The energy-minimized docked complexes and the apo S (S^closed^ and S^open^) structures, respectively, were subsequently refined by the 200 ns explicit solvent MD simulations. First, the steepest descent (SD) and CG methods were employed to remove the bad contact of initial structures. Second, each system was heated gradually from 0 to 310 K within 1.0 ns, with a positional restraint of 20.0 kcal mol^−1^ Å^−2^ on protein. Third, the systems were further equilibrated in a canonical ensemble (NVT) for 1.0 ns at 310 K with 10 kcal mol^−1^ Å^−2^ harmonic position restraints applied to the protein atoms. Finally, equilibration of the systems was carried out in an isothermal–isobaric ensemble (NPT, T = 310 K and P = 1 atm). MD snapshots were collected every 10 ps for post-MD analysis. All MD simulations were performed with periodic boundary conditions and the Newton-equations were integrated with a 2.0 fs time step using the Verlet algorithm. A cut-off distance of 10 Å was used for the van der Waals and electrostatic interactions [64]. Note: each complex was performed over two replicate simulations to ensure consistency.

### 3.5. Binding Free Energy Calculation

All values of binding free energies (Δ*G_bind_*) were calculated using molecular mechanics generalized born surface area (MM/GBSA) method [65], which has been successfully used to predict the binding affinities for ligand–protein interactions in the previous works [66]. Briefly, Δ*G_bind_* was estimated by using

Δ*G_bind_* = Δ*E_MM_* + Δ*G_GB_* + Δ*G_SA_* − *T*Δ*S* (1)

where the molecular mechanical contribution Δ*E_MM_* consists of internal energy (Δ*E_int_*), electrostatic (Δ*E_ele_*), and van der Waals (Δ*E_vdw_*). Δ*G_GB_* and Δ*G_SA_* are the polar and nonpolar contributions to solvation free energies. −*T*Δ*S* represents the entropic contribution [67,68]. All energy components were calculated on the basis of 300 snapshots extracted from 80~200 ns MD trajectories (150 snapshots evenly extracted from each MD trajectory), and the standard errors representing the estimation uncertainty were calculated based on 6 blocks (each block of 50 randomly selecting snapshots) [69,70].

### 3.6. Structural Analysis

The root-mean-square deviation (RMSD) of backbone atoms and the root-mean-square fluctuation (RMSF) of residues were analyzed by the cpptraj module in AmberTools 18 to measure the stability of the overall structure and the protein flexibility [71,72]. In addition, principal component analysis (PCA) was performed on the Cα atoms to understand the conformational changes upon ligand binding. Dynamic cross-correlation matrices (DCCM) were used to calculate average correlations between the motion of atoms in protein, which is composed of the fluctuation cross-correlations coefficient in the positions of Cα atoms during the MD simulation [73]. UCSF Chimera was used for structural plotting and visualization [74].

## 4. Conclusions

SARS-CoV-2 is the cause of respiratory distress syndrome, and there is a risk that COVID-19 will continue to worsen with the emergence of virus mutation [75]. In the current scenario, there is an urgent need to develop new potent drugs which can improve treatment of novel coronavirus. We modeled full-length structures of S^closed^ and S^open^ and found differences in the spatial orientation and structure of amino acids near HR1 and HR linker by comparing the dynamic structures of the two state conformations. Docking was carried out on the interface with large conformational differences to screen out SFN, isometheptene, urocanic acid, 1-octanol, allicin as potential inhibitors of S, where SFN with reported antioxidative and anticarcinogenic effect were selected for MD simulation according to geometric matching and energy analyses. SFN has high binding ability to S^closed^ and S^open^. We found that SFN can inhibit the counterclockwise rotation of the binding pocket—including the CH region, which is the process that the RBD ups to open state—and change the dynamic correlation between the fractional amino acids of HR1, HR linker, and HR2 of S^closed^ with the NTD, RBD region in S1 subunit. In addition, SFN can stabilize the conformation of the S^open^ and thus may inhibit the structural transition from the prefusion state to the postfusion state during membrane fusion. This study gives two more complete in situ structures of S protein and demonstrates the probable functioning mechanism of S^closed^ and S^open^. In addition, it also provides theoretical basis and application guidance for anti-SARS-CoV-2 drugs.

## Figures and Tables

**Table 1:** Docking results of top-five compounds (based on LibDock Score).

ZINC ID	Compound	Structure	Original Purpose	*LibDock* *Score*	
				S^closed^	S^open^
ZINC000002557133	Sulforaphane	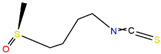	anticarcinogenic	70.62	78.16
ZINC000001683250	Isometheptene	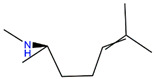	antihyperglycemic	69.26	58.46
ZINC000034633903	Urocanic acid	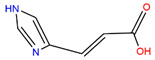	antihyperglycemic	68.20	68.90
ZINC000001532735	1-Octanol	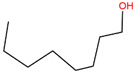	anti-cancer, antimicrobial	66.89	65.42
ZINC000004097409	Allicin	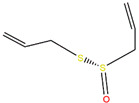	anti-inflammatory, anticancer	64.54	70.52

**Table 2:** Binding free energies (Δ*G_bind_*) and their components of docked complexes ^1^.

Complex	Δ*E_ele_*	Δ*E_vdw_*	Δ*G_SA_*	Δ*G_GB_*	Δ*G_bind_*
Sulforaphane-S^closed^	−10.90 ± 3.24	−30.92 ± 1.17	−4.51 ± 0.14	24.06 ± 3.28	−22.27 ± 1.12
Sulforaphane-S^open^	−8.70 ± 3.52	−24.34 ± 1.81	−3.62 ± 0.27	22.44 ± 1.98	−14.21 ± 2.53

^1^ All values are given in kcal mol^−1^, and behind “±” are their standard deviations (S.D.).

## Data Availability

Computational instructions and data of this work have been given in the main text and supporting information. Further information and requests may be directed and will be fulfilled by Zhiwei Yang (yzws-123@xjtu.edu.cn), the lead contact. Software used: BIOVIA Discovery Studio 3.1, https://www.3ds.com/ (accessed on 8 November 2022); Amber18, http://ambermd.org/ (accessed on 8 November 2022); AmberTools18, http://ambermd.org/AmberTools.php (accessed on 8 November 2022); UCSF Chimera 1.14, https://www.cgl.ucsf.edu/chimera/ (accessed on 8 November 2022).

## Supplementary Materials

The following supporting information can be downloaded at: https://www.mdpi.com/article/10.3390/ijms24076281/s1.
