# Supplementary material for: Identification of Potential Lead Compounds Targeting Novel Druggable Cavity of SARS-CoV-2 Spike Trimer by Molecular Dynamics Simulations
Source: Int J Mol Sci. 2023 Mar 27;24(7):6281. doi: 10.3390/ijms24076281 (PMC10094189; doi:10.3390/ijms24076281)
Supplement: Supplementary file 1 [file ijms-24-06281-s001.zip › ijms-2238476-supplementary.pdf]

International Journal of
Molecular Sciences

Article

Identification of Potential Lead Compounds Targeting Novel Druggable Cavity of SARS-CoV-2 Spike Trimer by Molecular Dy-Namics Simulations

Yizhen Zhao[1,†], Yifan Zhao[1,†], Linke Xie[1], Qian Li[2], Yuze Zhang[1], Yongjian Zang[1], Xuhua Li[1], Lei Zhang[1] and Zhiwei Yang[1,2,*]

[1] MOE Key Laboratory for Nonequilibrium Synthesis and Modulation of Condensed Matter, School of Physics, Xi'an Jiaotong University, Xi'an 710049, China
[2] School of Life Science and Technology, Xi'an Jiaotong University, Xi'an 710049, China
* Correspondence: yzws-123@xjtu.edu.cn
† These authors contributed equally to this work.

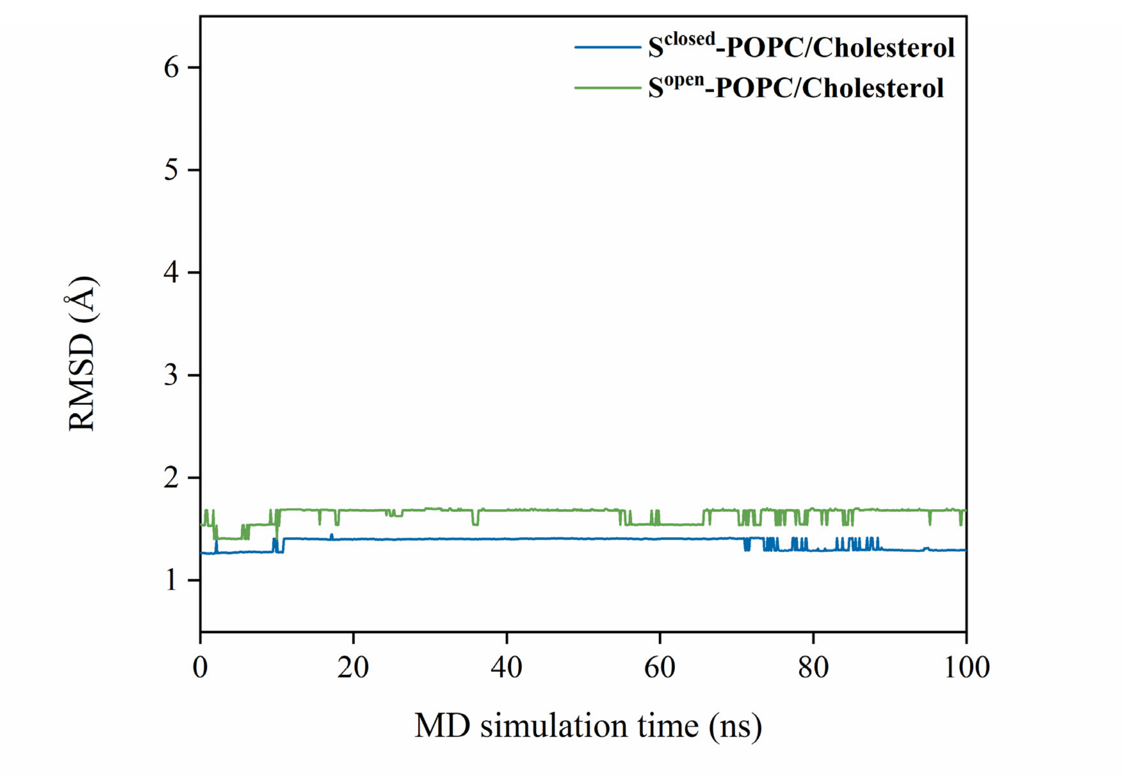

Figure S1. RMSD of S^{closed}-POPC/cholesterol and S^{open}-POPC/cholesterol systems.

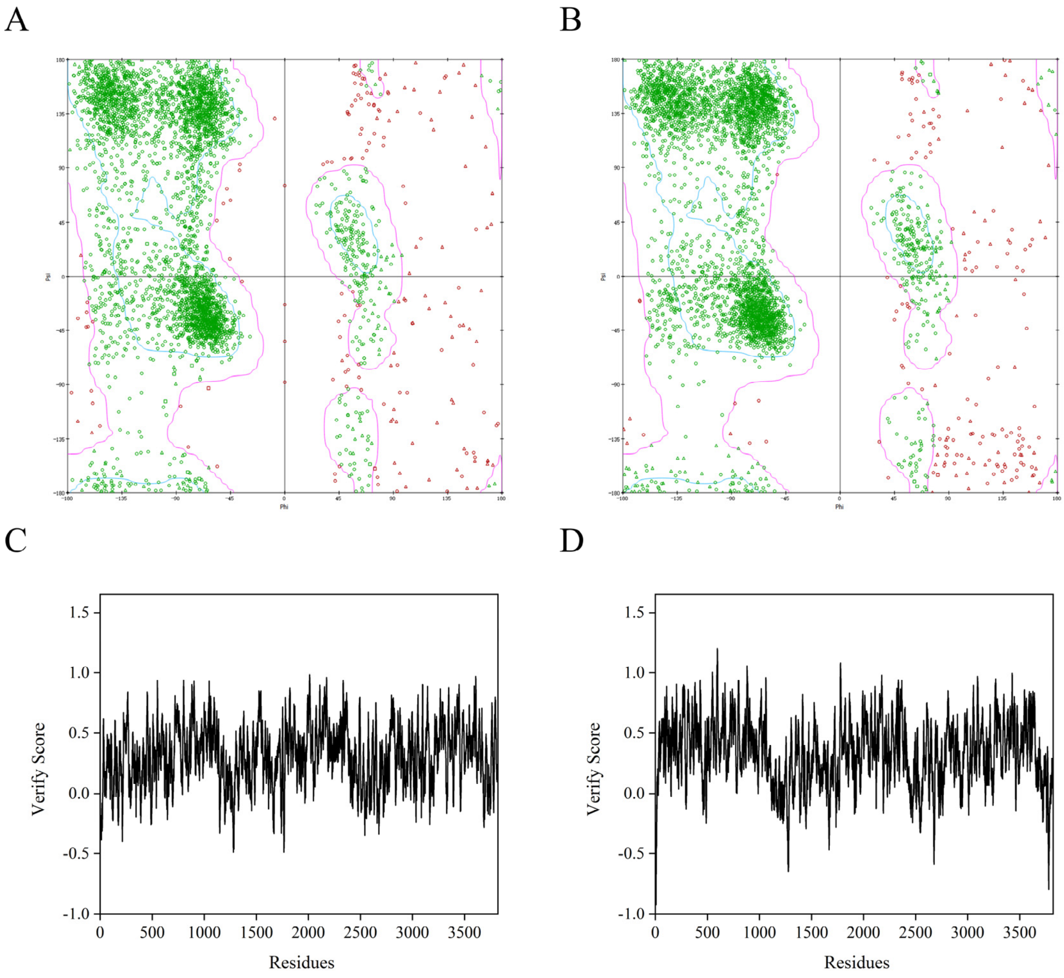

Figure S2. The Ramachandran plots of (**A**) S^{closed} and (**B**) S^{open}. The Profile-3D plots of (**C**) S^{closed} and (**D**) S^{open}. The homology modeling structures of S^{closed} and S^{open} are associated with 90% residues being in allowed region of Ramachandran plot (Procheck program) and 90% residues exhibiting reasonable folding (Profile-3D program).

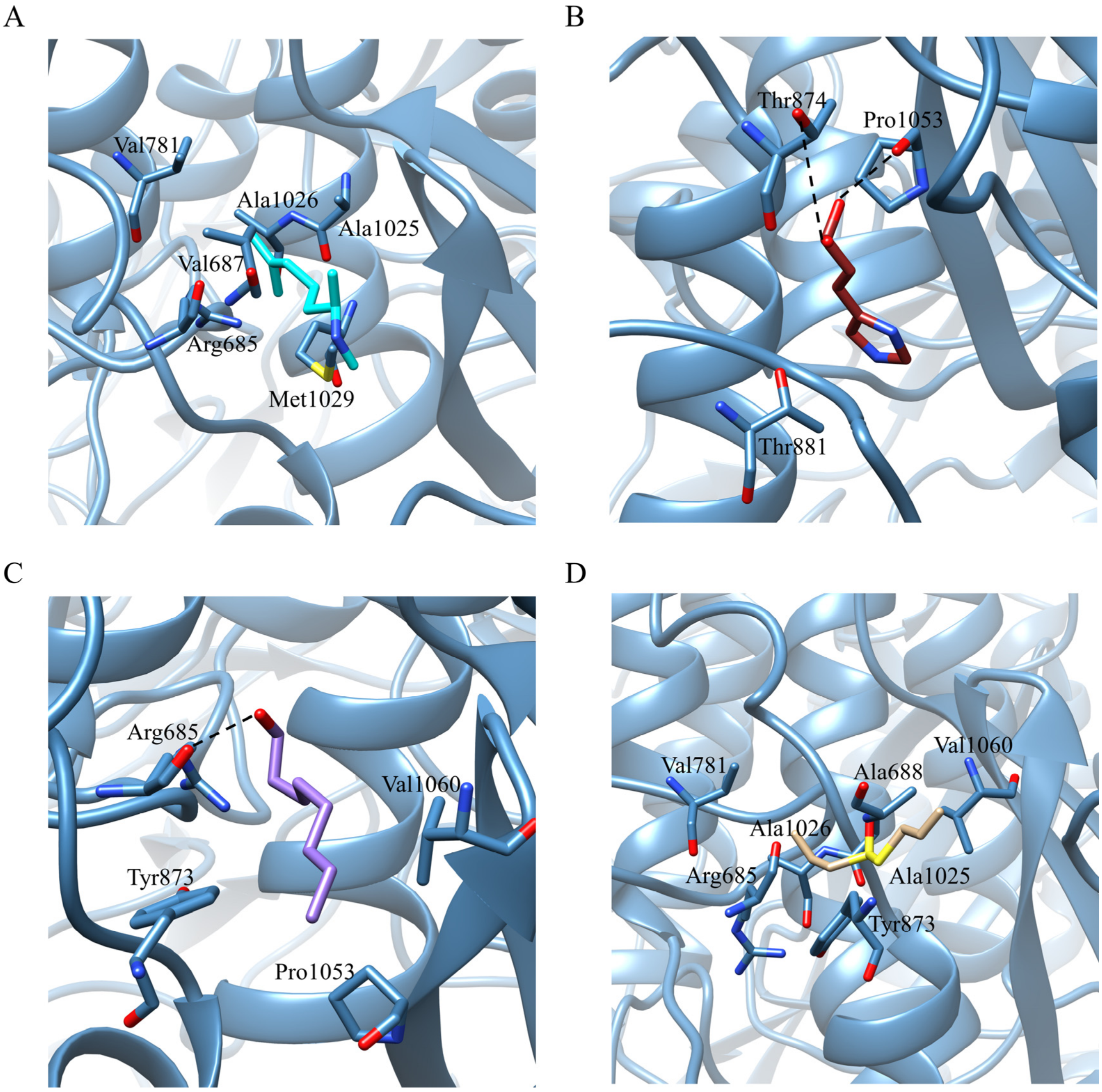

Figure S3. The binding interface of S^{closed} structure and (**A**) isometheptene, (**B**) urocanic acid, (**C**) 1-octanol and (**D**) allicin. The key residues are represented by stick models, and the important H-bonding interactions are labeled in the black lines.

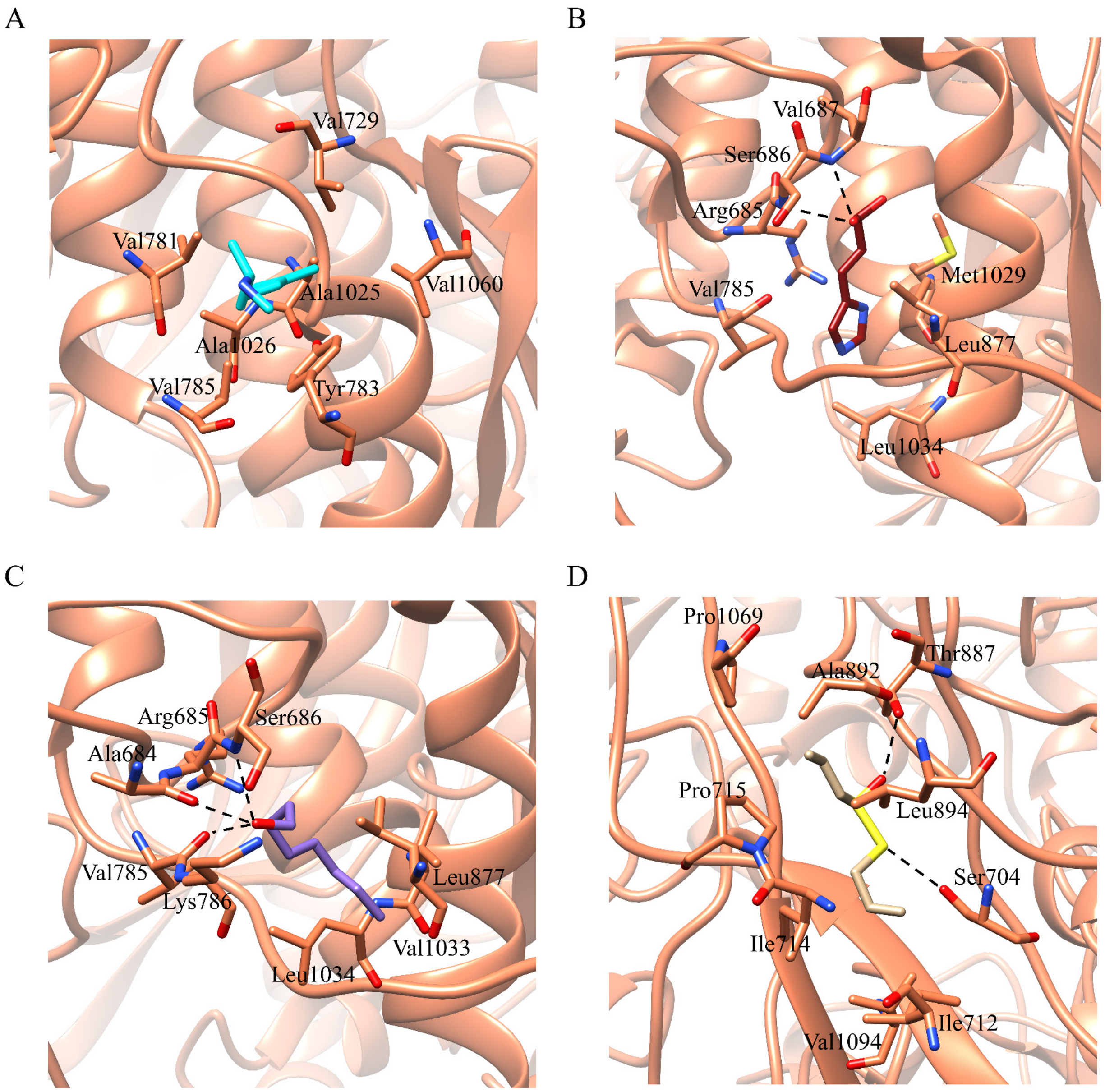

Figure S4. The binding interface of S^{open} structure and (**A**) isometheptene, (**B**) urocanic acid, (**C**) 1-octanol and (**D**) allicin. The key residues are represented by stick models, and the important H-bonding interactions are labeled in the black lines.

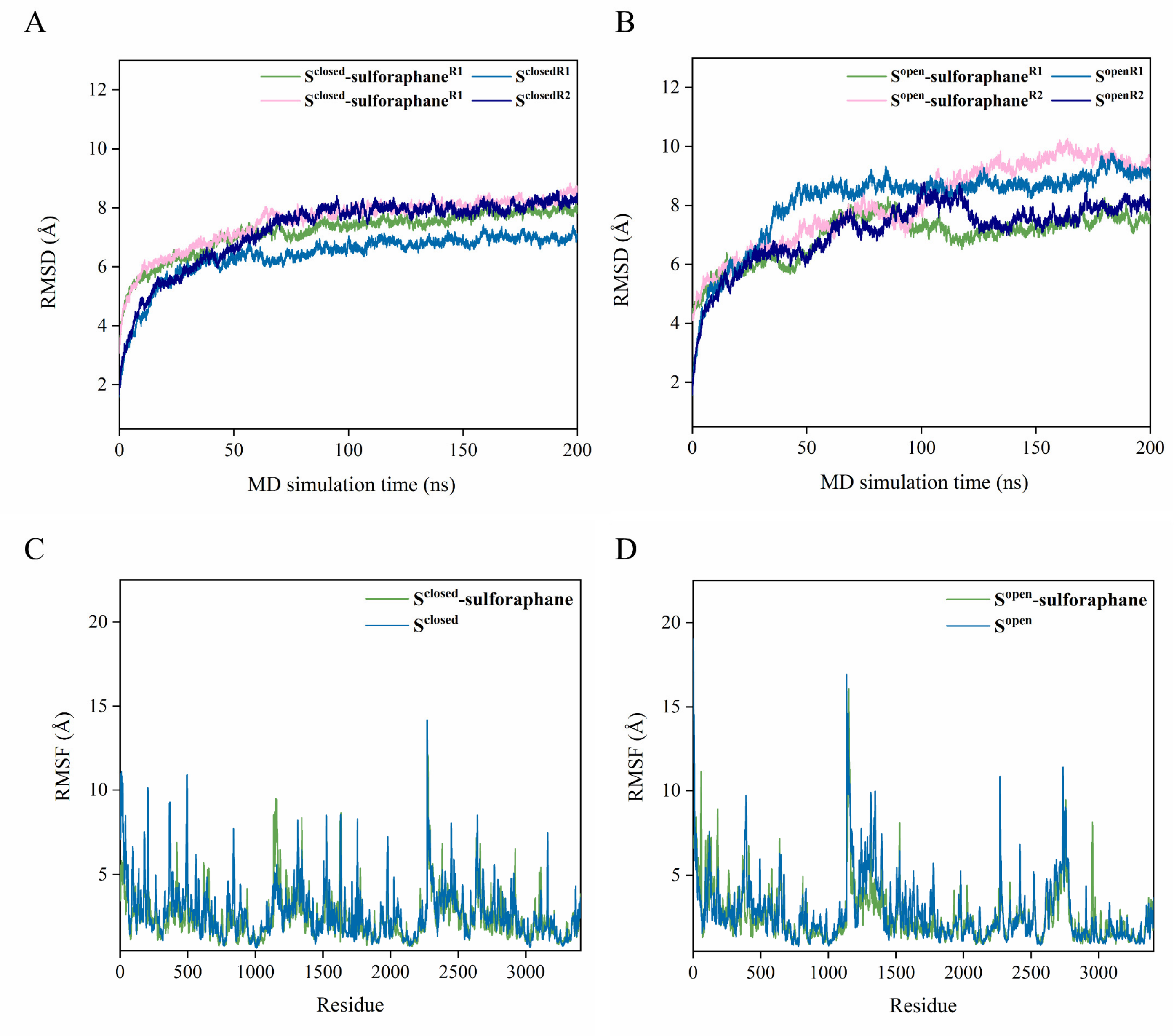

Figure S5. RMSD of various systems: (**A**) S^{closed} and (**B**) S^{open}. RMSF per residue for various systems: (**C**) S^{closed} and (**D**) S^{open}.
